# High expression of telomerase is an independent prognostic indicator of poor outcome in hepatoblastoma

**DOI:** 10.1038/sj.bjc.6602054

**Published:** 2004-07-27

**Authors:** E Hiyama, H Yamaoka, T Matsunaga, Y Hayashi, H Ando, S Suita, H Horie, M Kaneko, F Sasaki, K Hashizume, A Nakagawara, N Ohnuma, T Yokoyama

**Affiliations:** 1 Natural Science Center for Basic Research and Development, Hiroshima University, Hiroshima, Japan; 2 Department of Surgery, Graduate School of Biomedical Sciences, Hiroshima University, Hiroshima, Japan; 3 Japanese Study Group for Pediatric Liver Tumor, Japan

**Keywords:** human telomerase reverse transcriptase, telomerase, hepatoblastoma, prognosis, indicator, LightCycler

## Abstract

Telomerase, an enzyme related with cellular immortality, has been extensively studied in many kinds of malignant tumours for clinical diagnostic or prognostic utilities. Telomerase activity is mainly regulated by the expression of hTERT (human telomerase reverse transcriptase), which is a catalytic component of human telomerase. To evaluate whether the levels of *hTERT* mRNA provides a molecular marker of hepatoblastoma malignancy, we examined *hTERT* mRNA expression levels in the primary hepatoblastoma tissues by fluorescent RT–PCR using LightCycler technology and followed up the clinical outcomes in 63 patients listed in the Japanese Study Group of Pediatric Liver Tumor between 1991 and 2002. The *hTERT* mRNA expression was detected in 61 (96.8%) specimens and their expression levels ranged between 0.1/1000 and 745.1/1000 copies of PBGD gene that was used as an internal control. Among these cases, frozen 39 tumour samples and 14 adjacent noncancerous liver tissues were analysed for semiquantitative telomerase assay. In the 39 tumour samples, the levels of telomerase activity ranged between 0.11 and 2709 TPG and 12 (30.7%) had high telomerase activity (>100 TPG), whereas only nine of 14 noncancerous liver tissue samples showed telomerase activity which was less than 1.0 TPG. The levels of telomerase activity were significantly correlated with the levels of *hTERT* mRNA expression (*P*<0.001). The frequency of high *hTERT* mRNA expression and/or high telomerase activity did not significantly associate with the clinicopathological factors except for stage of disease. The prognosis of the patients with high *hTERT* mRNA expression was significantly worse than that of others (*P*<0.01), as was the patients with high telomerase activity (*P*<0.01). Multivariate analysis indicated that high levels of *hTERT* mRNA expression as well as telomerase activity are independent prognosis-predicting factors in patients with hepatoblastoma.

Hepatoblastoma is one of the common paediatric tumours and more than 70% of the tumours are diagnosed in children less than 2 years old (Weinberg and Finegold, 1983). This tumour, which is derived from hepatic precursor cells, is morphologically similar to immature hepatocytes and the prognosis of the patients is various. In the previous reports, tumour distribution, stage of tumour, and complete tumour resection were proposed to be the prognostic indicators in hepatoblastoma (Brown *et al*, 2000; Fuchs *et al*, 2002). The prognosis of children with hepatoblastoma has been improved significantly during the past two decades. Several multicentric trials such as the International Society of Pediatric Oncology (SIOP), United States-Intergroup, and our JPLT (the Japanese Study Group for Pediatric Liver Tumors) group studies, revealed that the successful reduction of large hepatoblastoma tumours by preoperative chemotherapy and complete resection are possible in many patients. In other instances, some tumours grow aggressively regardless of the use of preoperative chemotherapy. The latter tumours are considered to have high-grade malignancy. In advanced tumours with a low malignant grade, standard chemotherapeutic regimens are effective to reduce the primary tumour and to diminish metastatic tumours, resulting in patients' long survival, while new aggressive chemotherapy such as high-dose chemotherapy with stem cell transplantation is needed to cure the tumours with a high-grade malignancy (Nishimura *et al*, 2002). Thus, evaluation of the malignant grade of hepatoblastoma is necessary to improve the outcome of patients with advanced hepatoblastoma. Several molecular markers have been analysed to identify hepatoblastomas with high malignant potential: loss of heterozygosity (LOH) of chromosome 11p15.5, which is often affected in nephroblastoma and rhabdomyosarcoma in children, may contain a putative tumour suppressor gene for hepatoblastoma (Albrecht *et al*, 1994), but is unlikely to be a prognostic marker (Samuel *et al*, 1999; von Schweinitz *et al*, 2002). The mutation or deletion of the *β*-catenin gene exon 3 is frequently detected in hepatoblastoma, suggesting overactivation of the wingless/WNT signal pathway (Koch *et al*, 1999). This plays an important role in the pathogenesis of hepatoblastoma, but is not considered to be a good molecular marker to distinguish high-risk tumours from others (Takayasu *et al*, 2001; von Schweinitz *et al*, 2002).

In Japan, JPLT was opened to enrollment in 1991 and more than 150 patients were treated by JPLT protocols (Sasaki *et al*, 2002). The event-free survival (EFS) rate of patients with advanced stages was under 50%. Except for stage of disease, there are few markers to predict the prognosis of patients or to evaluate the malignant grade of hepatoblastoma. Elucidation of the useful prognosis-predicting factors is necessary to improve the prognosis of patients with hepatoblastoma.

Telomeres, which are specialised structures containing unique guanine-rich hexameric repeat sequences at the ends of human chromosomes (Blackburn, 1991), cannot be completely synthesised (referred to as the end-replication problem) with each cell division (Watson, 1972) and it is proposed that the loss of telomere eventually induces antiproliferative signals that result in cellular senescence (Shay, 1995). Telomerase is activated to maintain telomere length to compensate for the end-replication problem in germlines and immortal cells, but repressed in almost all human somatic cells. The activation of telomerase and the stabilisation of telomeres appear to be concomitant with the attainment of immortality in cancer cells (Harley *et al*, 1994; Kim *et al*, 1994; Shay, 1995). Telomerase activity has been found in approximately 85% of the cancer tissues examined, covering a large variety of cancer types including neuroblastoma, Wilms' tumour, and retinoblastoma (Hiyama *et al*, 1995a; Gupta *et al*, 1996; Dome *et al*, 1999; Hiyama and Hiyama, 2002). In some kinds of tumours, in which telomerase activity increases according to tumour progression, such as in neuroblastoma, non-small lung carcinoma, and colorectal cancer, the level of telomerase activity is a useful prognostic marker of the patients (Tatsumoto *et al*, 2000; Hiyama and Hiyama, 2002; 2003). Major components of telomerase are the RNA template (human telomerase RNA component: hTR) and the catalytic subunit (human telomerase reverse transcriptase: hTERT). hTR is expressed in the tissues with or without telomerase activity and is not correlated with the detection of telomerase activity, while hTERT expression is correlated with the detection of telomerase activity (Naito *et al*, 2001; Hiyama and Hiyama, 2002). Although *hTERT* transcripts show several splicing variants which have no telomerase activity (Wick *et al*, 1999), a system to detect full-length-*hTERT* mRNA alone has been developed.

To evaluate whether the levels of *hTERT* mRNA provides a molecular marker of hepatoblastoma malignancy, in the present study, we examined *hTERT* mRNA expression with this system and telomerase activity in hepatoblastoma specimens and compared the levels of their expression and the clinicopathological features and outcomes of the patients.

## MATERIALS AND METHODS

### Tissue samples

Hepatoblastoma tissue samples were obtained at surgery, immediately frozen, and stored at −80°C in the Tissue Bank of the JPLT or in the Hiroshima University Medical Hospital. In all, 63 tumours having total RNA samples available were enrolled in this study. The patients with these tumours were treated in the various hospitals or institutes under the framework of the JPLT between 1991 and 2001. Most patients were treated in the JPLT-1 study (Sasaki *et al*, 2002), which consisted of two different protocols: protocol 91A for patients with stage I or II hepatoblastoma and protocol 91B for patients with stage III or IV tumours. In these cases, 39 tumour samples with 14 corresponding normal liver tissues were stored at −80°C as frozen tissues and the remaining 24 samples were stored as total RNA samples.

### Clinical course and disease status

The clinicopathological parameters and outcomes for these 63 patients were analysed. The clinical stages of disease were determined at the time of initial biopsy or resection according to the classification of the Japanese Society of Pediatric Surgeons, which was based on the number of liver segments involved, the extent of local invasion, the extent of regional lymph node involvement, and the presence of distant metastases (Hata, 1990). The PRETEXT system (intrahepatic tumour extension) is based on hepatic surgical anatomy which is divided into four sectors, namely anterior and posterior sectors on the right and medial and lateral sectors on the left (Brown *et al*, 2000). Histological subtypes were diagnosed according to the classification of Haas *et al* and the Japanese Society of Pathology (Haas *et al*, 1989; Hata, 1990). Their criteria classified the tumours into four subtypes: a well-differentiated (fetal), a poorly differentiated (embryonal), immature (anaplastic) and other (including macrotrabecular pattern) types.

### Quantification of telomerase activity

Extraction of telomerase protein and evaluation of its activity were done by the TRAP (telomeric repeat amplification protocol) assay as described earlier (Kim *et al*, 1994; Piatyszek *et al*, 1995). Briefly, 50–100 mg of tumour or noncancerous liver tissues were homogenised in approximately 50–100 *μ*l of CHAPS lysis buffer. After 25 min of incubation on ice, the lysates were centrifuged at 16 000 **g** for 20 min at 4°C and the supernatant was rapidly frozen in liquid nitrogen and stored at −80°C. An aliquot of extract containing 0.5 *μ*g of protein was used for each assay. The levels of telomerase activity was measured using a commercial kit, the TRAPeze XL kit (Serological Co., Gaithersburg, MD, USA), which is a quantitative fluorescent-labelled PCR system for the estimation of relative telomerase activity with the use of a PCR internal control. The PCR product was measured in the fluorescent plate reader (Wallac, Perkin-Elmer, Wellesley, MA, USA) to detect the levels of fluorescein and sulphorhodamine by using the appropriate excitation and emission filters. The levels of telomerase activity were quantified by the ratio of the fluorescein intensity of the entire TRAP ladder to the sulphorhodamine intensity of the internal control after the correction of each fluorescent intensity for the negative control and the background, respectively, and were expressed as Total Product Generated (TPG) units.

### Quantification of *hTERT* mRNA expression

Using the acid-guanidium-phenol-chloroform method (Chromzynski and Sacchi, 1987), total cellular RNA was extracted. Quantitative detection of *hTERT* mRNA was performed with the LightCycler TeloTAGGG hTERT Quantification Kit (Roche Diagnostics, Mannheim, Germany) using the LightCycler instrument (Roche Molecular Systems, Alameda, CA, USA). For each sample, 100 ng of total RNA was prepared in a 20 *μ*l mixture containing 2 *μ*l of reaction mix, 0.1 *μ*l of reverse-transcriptase, and 2 *μ*l of hTERT or porphobilinogen deaminase (PBGD) detection mix. RT–PCR for the mRNA encoding the housekeeping gene *PBGD* was equally processed in a separate tube as a reference for relative quantification of *hTERT* mRNA expression. The mixture without template was examined as the negative control. These mixtures were reverse-transcribed for 10 min at 60°C, followed by denaturation (30 s at 95°C) and amplification of the 198-bp fragment of the *hTERT* mRNA sequence in 40 PCR cycles (0.5 s at 95°C, 10 s at 60°C, and 10 s at 72°C) using specific primers in a one-step RT–PCR. To establish a standard curve, five standards with *in vitro*-transcribed *hTERT* mRNA containing five different copy numbers were included in each experiment. The copy number of *hTERT* mRNA in each sample was normalised on the basis of its PBGD mRNA content according to the formula: *hTERT* mRNA expression level=*hTERT* mRNA copies/1000 PBGD mRNA copies.

### Statistical analysis

Correlations between the *hTERT* mRNA expression and telomerase activity levels or each of the other factors were analysed using Wilcoxon's *t*-test, χ^2^-test, or Fisher's exact test where appropriate. The overall survival curves for each group of patients were estimated by the Kaplan–Meier method and the resulting curves were compared using the Cox–Mantel test. Multivariate survival analysis using the Cox proportional hazard regression model was carried out to assess the independent contribution of each variable to disease-free survival. Differences were considered significant at *P*<0.05. A Computer program package (StatView 5.0; Abacus Concepts, Berkeley, CA, USA) was used for all of the statistical testing.

## RESULTS

### Clinicopathological findings (Table 1)

Among the 63 patients studied, the ages at diagnosis ranged between 0 and 13 years (mean 3 years and 6 months). They included nine stage I cases, 17 stage II, 13 stage IIIA, 10 stage IIIB, and 14 stage IV cases. Overall, 39 (61.9%) cases underwent curative surgery. Surgical resection was considered curative when no distant metastasis was evident and the clearance of cancer was complete as determined by standard histological analysis. The remaining 24 cases underwent noncurative surgery due to distant metastasis or extensive occupation of primary tumour. Totally, 34 cases underwent preoperative chemotherapy and all cases underwent postoperative chemotherapy.

In histological classification according to the pathological criteria of the Japanese Society of Pathology, 33 were classified as the well-differentiated type, 27 as the poorly differentiated type, two as immature and the remaining one case as other types. Serum levels of alpha-fetoprotein (AFP) ranged between 5 and 3 657 247 ng ml^−1^ and 56 cases showed more than 1000 ng ml^−1^ of AFP.

Among these patients, 11 died of disease, two showed recurrence of tumour and 50 are alive disease free. The survival periods ranged from 0 to 288 months (mean 74 months).

Out of 39 cases whose frozen tumour samples were available included six stage I cases, 13 stage II, five stage IIIA, eight stage IIIB, and seven stage IV cases. Among them 30 (75.9%) cases underwent curative surgery. Clinicopathological findings in these 39 cases were not significantly different from those in the whole cases (Table 1

**Table 1:** Patients and tumour characteristics

	**(Cases)**	***hTERT* mRNA (copies)**	**(Cases)**	**Telomerase activity (TPG)**
*Sex*				
Male	44	61.74±130.94	27	445.8±792.5
Female	19	37.48±90.80	12	403.4±809.6
				
*Age*				
0–11 months	12	27.28±62.94	7	458.1±990.0
12–23 months	18	57.85±97.72	12	722.3±1214.3
2–3 years	20	21.14±48.34	10	122.8±269.1
4–14 years	13	130.32±210.93	10	687.5±689.8
				
*PRETEXT*				
I	6	2.56±4.88	5	101.5±207.3
II	22	56.02±160.07	15	184.4±514.1
III	20	62.84±90.01	8	624.4±998.1
IV	11	65.55±106.82	9	579.7±1045.6
Unknown	4	25.76±50.09	2	630.1±890.6
				
*Stage*				
I	9	1.83±4.03	6	86.4±189.0
II	17	17.49±46.34	13	174.1±545.8
IIIA	13	39.63±61.47	5	333.1±706.7
IIIB	10	105.50±134.93	8	915.0±1072.0
IV	14	104.41±197.70	7	729.9±969.6
				
*Histology*				
Well	33	58.63±138.63	20	574.5±878.2
Poorly	27	51.21±96.00	17	312.8±704.3
Others	3	3.57±3.37	2	34.1±8.37
				
*Preoperative chemotherapy*				
Yes	34	63.61±149.14	19	486.6±802.7
No	29	40.18±65.60	20	381.6±789.7
				
*Curative surgery*				
Yes	39	56.91±130.29	30	397.4±758.8
No	24	46.19±96.98	9	550.3±914.6
				
*Prognosis*				
Survived with evidence-free	50	33.25±72.11	30	252.7±604.2
Recurrence/died of disease	13	128.11±207.25	9	1032.8±1046.0

).

### Levels of *hTERT* mRNA expression and telomerase activity in hepatoblastoma specimens

**Figure 1:**
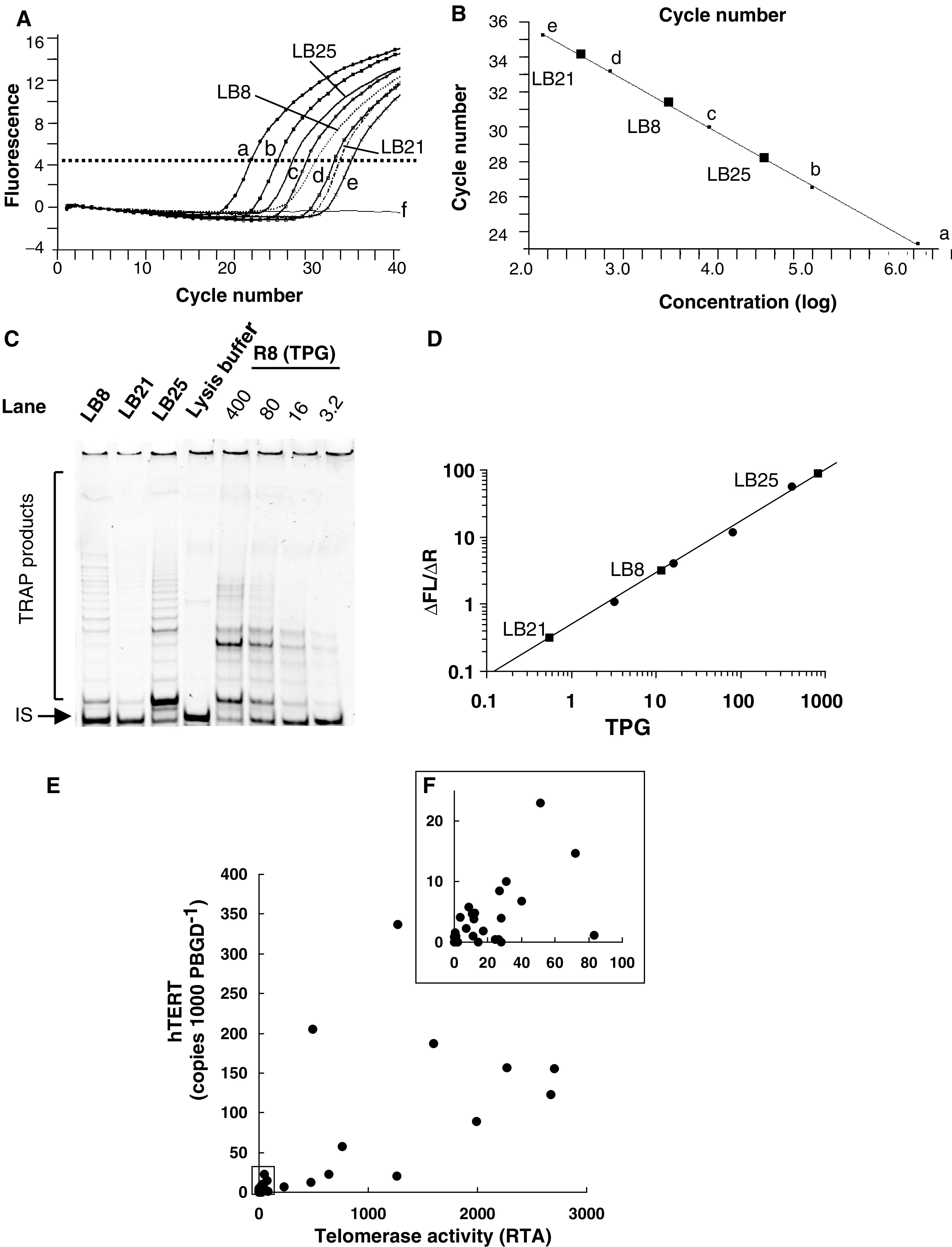
Detection of *hTERT* mRNA (**A**, **B**) and telomerase activity (**C**, **D**) and their relationship (**E**, **F**) in hepatoblastoma. (**A**) Amount of *hTERT* mRNA was measured by real-time RT–PCR analysis using LightCycler system in three representative hepatoblastoma samples (LB8, 21, and 25) with five external *hTERT* mRNA standards (a–e) and a negative control (f). (**B**) *hTERT* mRNA levels of three representative samples were calculated by the standard curve of the external *hTERT* RNA standards (a–e). (**C**) Detection of telomerase activity was done using the TRAPeze XL kit (Serological Co., Gaithersburg, MD, USA), which is a quantitative fluorescence-labelled PCR system for the estimation of relative telomerase activity with the use of a PCR internal control (IS). Positive controls included serial diluted control template (R8), oligonucleotides with eight telomeric repeats AG(GGTTAG)_7_, to produce a standard curve. (**D**) The levels of telomerase activity were quantified by the ratio of the fluorescein intensity (ΔFL) of the entire TRAP ladder to the sulphorhodamine intensity (ΔR) of the internal control, and were expressed as Total Product Generated units (TPG). Levels of telomerase activity in the three representative samples (LB8, 21, and 25) were calculated by the standard curve using ΔFL/ΔR of the external standard R8. The levels of telomerase activity in LB8, LB21, and LB25 were calculated as 31.1, 0.37, and 761.7 TPG, respectively. (**E**, **F**) The correlation between the levels of *hTERT* mRNA expression normalised to the internal control PBDG and those of telomerase activity in overall 39 hepatoblastoma samples (**E**) and those with low telomerase activity (**F**). There is a significant correlation between these two parameters (*P*<0.0001).

Among the 63 primary hepatoblastoma specimens obtained, 58 (92%) specimens displayed apparent *hTERT* mRNA expression using the quantitative *hTERT* mRNA expression assay (Figure 1A–C

). The levels of *hTERT* mRNA expression ranged from 0.008 to 745.1 (mean 49.5) copies 1000 copies^−1^ of the *PBDG* mRNA. In these 58 cases, 24 (38.1%) showed high levels of *hTERT* mRNA expression (more than 10 *hTERT* mRNA copies 1000 copies^−1^ of the *PBDG* mRNA). In the 14 noncancerous liver specimens examined, only two samples derived from two patients under 1-year old showed *hTERT* mRNA expression, but their levels were low (0.42 and 0.78). Among these cases, telomerase activity was examined in 39 cases. Using quantitative TRAP assay (Figure 1D), telomerase activity ranged between 0.11 and 2669 TPG (mean 432.7 TPG). As previously described (Tatsumoto *et al*, 2000), more than 100 TPG was defined as high telomerase activity. Overall, 12 cases (30.8%) showed high telomerase activity. Figure 1E shows the correlation between *hTERT* mRNA expression levels and telomerase activity levels. There was a significant correlation between these two expression levels (*γ* =0.87, *P*<0.01).

### Levels of *hTERT* mRNA expression or telomerase activity and the clinicopathological features of the patients

Table 1 shows the correlation between *hTERT* mRNA expression or telomerase activity levels and the clinicopathological features of the patients. Regarding age at diagnosis, the levels of *hTERT* mRNA expression and of telomerase activity were high in the elder patients, but not significantly. In histological classification, there was no significant difference of the levels of *hTERT* mRNA expression or telomerase activity between well- and poorly differentiated types. In PRETEXT classification, the levels of *hTERT* mRNA expression increased in PRETEXT 2, 3, and 4 tumours but not significantly (*P*=0.116). The levels of telomerase activity in the PRETEXT 2, 3, and 4 tumours were significantly higher than in the PRETEXT 1 tumours (*P*=0.025). The levels of *hTERT* mRNA expression and telomerase activity significantly increased in advanced stages (stages IIIA, IIIB, and IV, *P*=0.0146 and 0.0234, respectively) and in tumours with distant metastasis (stage IV *vs* others), but not significantly. The levels of *hTERT* mRNA expression (mean 49.9, *n*=22) and telomerase activity (mean 369.1 TPG, *n*=14) in tumours obtained after preoperative chemotherapy did not significantly differ from those in tumours obtained without any therapies (mean 54.4, *n*=42 and mean 435.4, TPG, *n*=25, respectively). There was no significant correlation between the levels of serum AFP and *hTERT* mRNA expression or telomerase activity.

### Correlation between *hTERT* mRNA expression and prognosis of the patients

**Figure 2:**
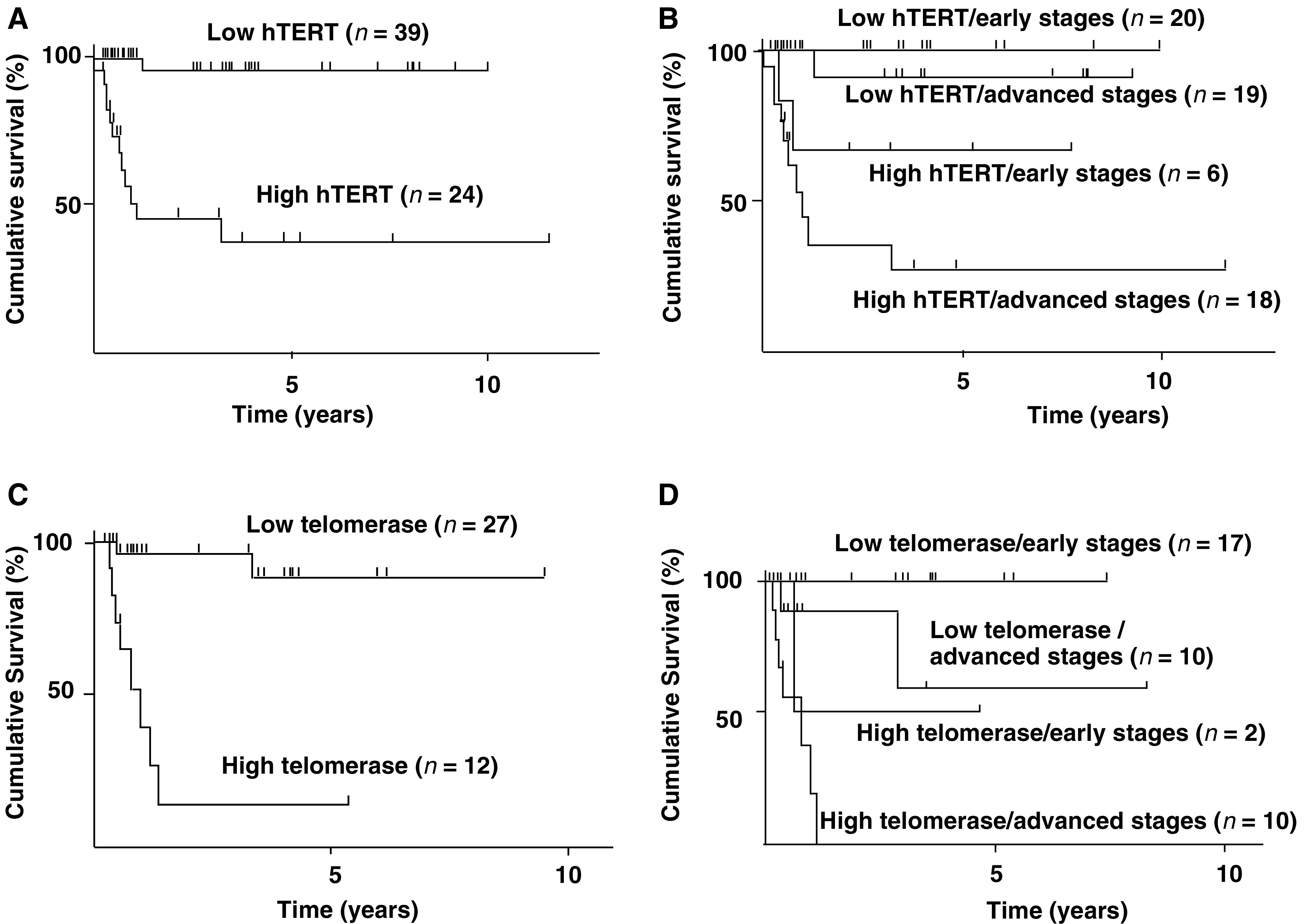
Kaplan–Meier cumulative survival spots for patients with hepatoblastoma. (**A**) Survival according to the levels of *hTERT* mRNA expression. High *hTERT*: *hTERT* mRNA ⩾10 copies 1000 copies^−1^ of the *PBDG* genes, low *hTERT*: *hTERT* mRNA <10 copies 1000 copies^−1^ of the *PBDG* genes. The patients with tumours with high *hTERT* mRNA expression showed significantly worse survival (*P*<0.0001). (**B**) Survival according to the levels of *hTERT* mRNA expression and stages. Early stages: I and II, advanced stages: III and IV in the stage classification of the Japanese Society of Pediatric Surgeons. Patients having advanced tumours with high *hTERT* mRNA expression showed significantly worse survival (*P*<0.0001). (**C**) Survival according to the levels of telomerase activity. High telomerase: TPG value ⩾100, low telomerase: TPG value <100. The patients having tumours with high telomerase activity showed significantly worse survival (*P*<0.0001). (**D**) Survival according to the levels of telomerase activity and stages. The patients having advanced tumours with high telomerase activity showed significantly worse survival (*P*<0.0001).

The median follow-up in the series of patients examined was 74 months (range, 1–288 months). Kaplan–Meier event-free survival (EFS) curves of all patients (Figure 2A

) show that the 10-year EFS rate in the patients with high *hTERT* mRNA expression (⩾10 copies 1000 copies^−1^ of the *PBDG* gene) was 38%, while that in the remaining patients was approximately 90%. The prognosis of the patients with high expression of *hTERT* was significantly worse than that of other patients (*χ*^2^=23.40, *P*<0.0001). Since the levels of *hTERT* mRNA were significantly correlated with advanced stages of tumour, the correlation between *hTERT* mRNA expression and prognosis was examined in tumours in early stages (stage I or II), and those in advanced stages (stage III or IV), separately (Figure 2B). The prognosis of the patients with high levels of *hTERT* mRNA expression was significantly poor in advanced tumours (*χ*^2^=26.03, *P*<0.0001). In 26 patients with early tumours, all 20 patients with low levels of *hTERT* mRNA expression are alive disease free and two out of six patients with high levels of *hTERT* mRNA expression showed poor prognosis (*χ*^2^=3.291, *P*=0.046).

### Correlation between the levels of telomerase activity and prognosis of the patients

This study attempted to determine the effect of telomerase activity and *hTERT* mRNA expression on the prognosis of patients with hepatoblastoma. Telomerase activity was investigated in only 39 cases because frozen tumour tissue was unavailable in the remaining 24 cases. Kaplan–Meier EFS curves of these 39 patients (Figure 2C) show that the 10-year EFS rate in the patients with high telomerase activity (TPG ⩾100) was approximately 40%, while that in the remaining patients was approximately 90%. The prognosis of the patients with high telomerase activity (TPG ⩾100) was significantly worse than that of other patients (*P*=0.0003). Since the levels of telomerase activity were significantly correlated with advanced stages of tumour, the correlation between telomerase activity and prognosis was examined in the tumours in early stages (stage I or II) and those in advanced stages (stage III or IV), separately (Figure 2D). The prognosis of the patients with high telomerase activity was significantly poor in advanced tumours (*χ*^2^=27.12, *P*<0.0001). In early tumours, one of two patients with high telomerase activity showed poor prognosis, while all patients with low telomerase activity are alive disease free.

### Prognostic factors

By univariate analysis, we analysed clinical parameters such as PRETEXT classification, distant metastasis, stage classification, serum levels of AFP, histological classification, preoperative chemotherapy, and total surgical resection, for the correlation with prognosis of the patients. PRETEXT 2, 3, and 4 tumours and the tumours with distant metastasis showed poorer prognosis, but not significantly. On the other hand, advanced stages (stages 3 and 4) were significantly correlated with poor prognosis of the patients (*P*=0.022). Thus, both telomerase activation and advanced stages were correlated with poor prognosis of patients.

To identify which independent factors had a significant influence on survival, multivariate survival analysis using the Cox proportional hazard regression model was performed. In this multivariate analysis, we assessed the prognostic value for event-free survival of the parameters that were significant in univariate analysis: stage and *hTERT* mRNA expression. For this multivariate analysis, variables with *P*-value lower than 0.30 in the univariate analysis were also selected: gender, age at diagnosis, curative surgery, stage/PRETEXT classification, histology, and hTERT/ telomerase activity. As stage classification was significantly associated with PRETEXT and distant metastasis, we used stage classification in this multivariate analysis. As the levels of *hTERT* mRNA expression were significantly correlated with the levels of telomerase activity, and telomerase activity could not be analysed in 24 cases, we analysed these two factors separately in different multivariate analysis sheets. In the multivariate analysis including *hTERT* mRNA expression and the other five factors for 63 cases, *hTERT* mRNA and poorly differentiated histology were independent predictors of EFS. The hazard ratios were 50.0 (95% confidence interval of 5.07−492.9, *P*=0.0008) and 5.11 (95% confidence interval of 1.16−22.5, *P*=0.031). In the multivariate analysis including telomerase activity and the other five factors for 39 cases, the level of telomerase activity was also an independent predictor of EFS. The hazard ratio of telomerase activity levels was 17.7 (95% confidence interval of 2.16−120.1, *P*=0.0032). In advanced stages, the hazard ratios for *hTERT* mRNA and telomerase activity levels were 9.221 (*P*=0.043) and 5.248 (*P*=0.188), respectively.

## DISCUSSION

Clinical investigation revealed that the prognosis of children with hepatoblastoma correlates with multifocal growth in the liver, invasion of blood vessels, distant metastasis, and either very low or high levels of serum AFP (von Schweinitz *et al*, 1997; Brown *et al*, 2000). Survival rates of children with more than two of these factors were less than 10%. Thus, these findings discriminate a subgroup of hepatoblastoma with more aggressive biological properties, which correlate with a poor prognosis. However, these factors are not sufficient to predict the prognosis of children with hepatoblastoma. Recently, high-dose chemotherapy with stem cell transplantation has become effective in some patients with aggressive hepatoblastoma with metastasis (Nishimura *et al*, 2002). Thus, to identify such high-risk patients with hepatoblastoma, we need additional useful prognostic markers for evaluation of aggressive biological properties.

Telomerase activity has been reported in many kinds of malignant tumours, including gastric cancer (Hiyama *et al*, 1995b), hepatocellular carcinoma (Nouso *et al*, 1996; Nakashio *et al*, 1997), pancreatic cancer (Hiyama *et al*, 1997b; Iwao *et al*, 1997), and colorectal cancer (Chadeneau *et al*, 1995; Naito *et al*, 2001). Approximately 80–90% of these malignant tumours showed telomerase activity (Shay and Bacchetti, 1997). In some kinds of tumours, high telomerase activity has been reported as a marker of tumour aggressiveness and poor prognosis (Hiyama *et al*, 1995a; 1995b; Shay *et al*, 1997; Marchetti *et al*, 1999). In childhood tumours, telomerase activity and *hTERT* mRNA expression were also detected in a majority of cases of neuroblastoma, retinoblastoma, and nephroblastoma. In neuroblastoma, we have already reported a significant correlation between high telomerase activity and poor outcomes of patients (Hiyama *et al*, 1995a; 1997a). In retinoblastoma, telomerase activity was detected in about 50% of the tumours and such tumours showed a high recurrence rate (Gupta *et al*, 1996). In the present study, *hTERT* mRNA expression and telomerase activity were correlated with poor prognosis of patients, indicating these factors are useful prognosis-predicting factors in hepatoblastoma. Thus, activation of telomerase may correlate with malignant potential in most childhood malignant tumours including hepatoblastoma, neuroblastoma, and retinoblastoma.

This is the first report to show an association between the levels of *hTERT* mRNA expression or telomerase activity and patient prognosis in hepatoblastoma. In the multivariate analysis, activation of telomerase, stage of disease, and histological type were significantly correlated with the outcome of patients. In these three independent parameters, the risk of *hTERT* mRNA or telomerase activation was highest, indicating that telomerase reactivation is the most useful prognosis-associating factor in hepatoblastoma. In the present study, four (15.4%) of the 26 cases with early stage hepatoblastoma showed recurrence of tumours, and all four cases showed high telomerase activity (TPG ⩾100 or *hTERT* mRNA ⩾100 copies). In contrast, three (16.7%) of the 18 advanced cases with high telomerase activity remain disease-free. Since all stage 4 cases underwent different chemotherapeutic regimen in the JPLT study (Sasaki *et al*, 2002), one explanation for this result is that the high-dose chemotherapy might have been effective in preventing recurrence in these four early cases. Thus, in early stage tumours, selection of patients for high-dose chemotherapy based on high telomerase activity (TPG ⩾100) might be an effective method to improve the prognosis of this category of patient. Moreover, the exclusion of low-risk patients from postoperative chemotherapy could spare some of its serious side effects. In advanced hepatoblastoma with low malignancy, complete resection and chemotherapy should be performed, but in such tumours with high malignancy, complete resection and chemotherapy might be insufficient and new aggressive strategies should be implemented. The observations in our study suggest that telomerase inhibition is an effective strategy for the reversal of tumour growth. Since most somatic cells do not have detectable telomerase activity and telomerase shows a tumour-specific expression in general, telomerase is an important target for new anticancer therapy. A number of different approaches have been developed for telomerase inhibition in human cancer. Different components and type of inhibitors targeting various regulatory levels have been regarded as useful telomerase inhibitors and seem to be most efficient when combined with conventional chemotherapy (Saretzki, 2003). Telomerase inhibition, which may be involved in triggering apoptosis, may be a new strategy for curing hepatoblastoma in the future.

In the present study, we analysed the clinical variables of hepatoblastoma cases, but did not find significant correlation between the levels of *hTERT* mRNA or telomerase activity and these variables except for PRETEXT system and disease-stage. It is well-known that prognosis of the cases with pure-foetal histology is good (Finegold, 2002). In the present study, we had only two pure-foetal subtypes in 33 well-differentiated tumours. Although the levels of *hTERT* mRNA or telomerase activity in them were relatively low, further study with large number of this subtype is necessary to analyse statistically.

Some noncancerous childhood liver tissues showed low levels of telomerase activity and *hTERT* mRNA expression. In childhood liver tissue infiltrating lymphocytes, multipotential stem cells, and their daughter cells might have telomerase activity, resulting in positive results by the contamination of lymphocytes and stem cells with telomerase activity. Recently, it was reported that telomere maintenance by the existence of telomerase activity is necessary for the proliferation of normal human cells (Masutomi *et al*, 2003). Thus, low levels of telomerase activity may reflect the proliferation of normal hepatocytes in children. To solve this false-positive problem, *in situ* evaluation is necessary to analyse the origin of telomerase expression in clinical samples using *hTERT* mRNA ISH (Chou *et al*, 2001; Kumaki *et al*, 2001; Kotoula *et al*, 2002) or hTERT immunohistochemistry (Yasui *et al*, 1999; Hiyama *et al*, 2001).

In summary, we show that an increased level of *hTERT* mRNA expression or telomerase activity is a prognostic indicator of poor outcome in patients with hepatoblastoma, independent of disease stage and histological classification. Although it would need large series to clarify the correlation between clinical variables and the levels of *hTERT* mRNA or telomerase activity, high telomerase activity may stratify patients that are likely to have cancer recurrence requiring postoperative aggressive chemoadjuvant therapy, or, in the future, telomerase-targeting therapy.
